# Current Practices and Emerging Therapies to Optimize Heart Failure Management in Cardiac Sarcoidosis: A Systematic Review

**DOI:** 10.7759/cureus.66515

**Published:** 2024-08-09

**Authors:** Aadi R Palvia, Avneet Kaur, Gibran A Azeez, Mounika Thirunagari, Nazeefa Fatima, Abhinav Anand, Sondos T Nassar

**Affiliations:** 1 Internal Medicine, California Institute of Behavioral Neurosciences & Psychology, Fairfield, USA; 2 Pathophysiology, St George's University, St George's, GRD; 3 Internal Medicine, Davao Medical School Foundation, Davao City, PHL; 4 Clinical Research, California Institute of Behavioral Neurosciences & Psychology, Fairfield, USA; 5 Medicine and Surgery, Jordan University of Science and Technology, Amman, JOR

**Keywords:** azathioprine treatment, oral methotrexate, heart transplantation, tnf alpha inhibitor, immunosuppresive therapy, corticosteroid therapy, systemic sarcoidosis, isolated cardiac sarcoidosis, heart failure, management of heart failure

## Abstract

Cardiac sarcoidosis (CS) is a distinctive manifestation of sarcoidosis, a multisystemic inflammatory disorder that is characterized by non-necrotizing granulomas. CS can lead to arrhythmias, heart failure (HF), and sudden cardiac death. The diagnosis of CS involves imaging in the form of a two-dimensional echocardiogram, cardiac magnetic resonance imaging (MRI), an 18-fluoro-deoxyglucose positron emission tomography (FDG-PET) scan, and an endomyocardial biopsy. Treatment of CS entails corticosteroids, immunosuppressive agents, monoclonal antibodies, and, in advanced cases, heart transplantation (HTx). This systematic review follows Preferred Reporting Items for Systematic Reviews and Meta-Analyses (PRISMA) 2020 guidelines, focusing on HF in sarcoidosis patients. Eligibility criteria include recent (2019-2024) research papers on sarcoidosis-induced heart failure, excluding other causes. The databases searched were PubMed, Google Scholar, and ScienceDirect. From 36,755 initial articles, 2,060 remained after filtering, and 17 were selected for quality assessment. Based on quality assessment, 11 studies were included in the final review. In CS, a variety of treatment strategies can be implemented. Corticosteroids are the first-line therapeutic options, and in the majority of cases, they are very successful in controlling the disease progression. Immunosuppressive agents like methotrexate and azathioprine are used to avoid long-term steroid use. Both corticosteroids and immunosuppressives act by reducing inflammation and preventing myocardial scarring. Biological agents like infliximab and adalimumab prevent disease progression by targeting specific inflammatory pathways and are used in refractory cases. Regular HF management drugs like angiotensin-converting enzyme (ACE) inhibitors, angiotensin receptor blockers (ARBs), sodium-glucose transport protein 2 (SGLT2) inhibitors, beta-blockers, and diuretics help in optimizing cardiac function. In severe cases, a left ventricular assist device (LVAD) may be required. The ultimate treatment for end-stage CS is HTx, which has to be supplemented with a strong, individualized regimen of glucocorticoids and immunosuppressives to avoid graft rejection and to control sarcoidosis. Due to a lack of standard protocols for management and limited knowledge about CS, the ideal treatment of HF is still a matter of debate. Hence, further research and clinical trials need to be performed to optimize patient outcomes.

## Introduction and background

Sarcoidosis is a multisystemic disorder with an uncertain etiology that causes non-necrotizing granulomas to infiltrate numerous organs. African Americans and Scandinavians are more likely to have the condition, which has a greater prevalence in individuals under 50, especially in the 25-40 age range, attaining a subsequent peak in women over 50. The yearly incidence of sarcoidosis varies between 2.3 and 11 per 100,000 people [1]. According to autopsy data, cardiac involvement is seen in up to 25% of individuals with systemic sarcoidosis, and this is linked to a poorer prognosis. The degree and location of the heart involvement determine the symptoms of cardiac sarcoidosis (CS), which can range from asymptomatic illness to serious conditions such as heart failure (HF), high-grade atrioventricular block, or ventricular tachycardia (VT) [2]. Patients with sarcoidosis had a much greater incidence of HF than individuals without the disease (240 versus 12 per 1000 patient-years, respectively; P<0.001). The risk of developing HF was significantly greater in individuals with sarcoidosis upon adjusting for age, sex, race/ethnicity, and comorbidities. Compared to other known risk factors, sarcoidosis showed the highest relative risk of HF, with a hazard ratio of 11.2 (P<0.01) [3]. Initial assessments using a chest computed tomography (CT) scan and X-rays are part of the diagnostic approach for CS. The use of speckle-tracking echocardiography has allowed for more nuanced monitoring than conventional echocardiography. With its excellent diagnostic accuracy, cardiac magnetic resonance imaging (MRI) uses late gadolinium enhancement and native T1 mapping to identify fibrosis and edema. Biopsies are guided by 18-fluoro-deoxyglucose positron emission tomography (FDG-PET) scans, which identify areas of metabolic activity, and by hybrid PET/MRI scanners, which efficiently integrate both modalities. The gold standard for diagnosis is still endomyocardial biopsy, with imaging-guided methods improving accuracy [4].

The treatment of CS is challenging, and the optimal course of action remains a subject of ongoing debate. Experts advise initiating immunosuppressive treatment for cardiac involvement, such as arrhythmias and HF symptoms. Although steroid-sparing regimens are recommended, trustworthy, long-term therapeutic guidelines are lacking. It's uncertain if immunosuppressives are often beneficial for symptoms that are not life-threatening. Research is currently ongoing to determine the best ways to assess therapy response, treatment duration, and dose recommendations [5]. To reduce myocardial inflammation and treat the implications of cardiac damage and scarring, effective medicines are critical in the management of CS. Prednisone is frequently used as a first-line therapy, with dosage and other immunomodulators modified according to the severity of clinical symptoms. Second-line treatments, such as methotrexate or azathioprine, are used when corticosteroids are inadequate or must be lowered owing to harmful side effects. Biologic drugs such as infliximab, adalimumab, and rituximab are third-line therapies that require patient screening for infection prior to their use [6]. Currently, trials are being conducted to investigate novel therapy alternatives and enhance treatment procedures. Severe occurrences, such as fulminant myocarditis associated with CS, may require strong immunosuppression along with mechanical assistance. Left ventricular assist device (LVAD) and cardiac transplantation are options for individuals with terminal HF; studies have shown that post-transplant results for people with and without CS are comparable and that CS recurrence in transplanted hearts is uncommon [6]. The prognosis for CS varies significantly by type, with isolated CS showing poorer event-free survival (63.0% adverse events) compared to systemic sarcoidosis (12.7%). It is theorized that delayed diagnosis and treatment contribute to worse outcomes in isolated CS, while early corticosteroid therapy in systemic cases improves left ventricular function. Early diagnosis and treatment are critical for improving outcomes in CS, particularly in isolated cases, highlighting the need for further research into optimal management strategies [7].

## Review

Methods

The Preferred Reporting Items for Systematic Reviews and Meta-analyses (PRISMA) 2020 guidelines have been followed in this systematic review [8]. Participants are patients diagnosed with sarcoidosis and presenting with HF. Interventions include various therapeutic strategies aimed at managing HF in sarcoidosis. Finally, the outcome included an assessment of improvement in cardiac function and a reduction in HF symptoms. Table 1 illustrates the inclusion and exclusion criteria that were introduced.

**Table 1:** Specifics of the inclusion and exclusion criteria RCT: Randomized controlled trial

	Inclusion criteria	Exclusion criteria
Research paper	Heart failure due to sarcoidosis	Heart failure due to other causes except sarcoidosis
Publication date	Last five years (2019-2024)	More than five years or before 2019
Literature	Published literature	Author letters, conference abstracts, books, gray literature, non-published literature
Study types and designs	Narrative reviews, systematic reviews, meta-analyses, case reports, observational, RCT, cohort studies	Nil
Population	People with cardiac sarcoidosis	Those not affected by cardiac sarcoidosis
Sex	Both female and male	Nil
Language	English	All languages other than English
Text availability	Free full text only	Abstracts, paid full text

Using the databases ScienceDirect, Google Scholar, and PubMed, a methodical search was carried out. The databases were last searched in July 2024. Heart failure and sarcoidosis were the terms entered into the search engines, and the medical subject headings (MeSH) approach was employed in PubMed. Table 2 provides more details about the search strategy used in PubMed and other databases.

**Table 2:** Search strategy used to obtain literature for the study MeSH: Medical subject headings

Databases	Search strategy	Number of articles before filters	Filters	Search result
PubMed	Heart Failure OR ("Heart Failure/diagnosis"[Mesh] OR "Heart Failure/diagnostic imaging"[Mesh] OR "Heart Failure/etiology"[Mesh] OR "Heart Failure/history"[Mesh] OR "Heart Failure/pathology"[Mesh] OR "Heart Failure/physiopathology"[Mesh] ) AND Sarcoidosis OR ( "Sarcoidosis/complications"[Mesh] OR "Sarcoidosis/diagnosis"[Mesh] OR "Sarcoidosis/epidemiology"[Mesh] OR "Sarcoidosis/history"[Mesh] OR "Sarcoidosis/mortality"[Mesh] OR "Sarcoidosis/pathology"[Mesh] OR "Sarcoidosis/physiopathology"[Mesh] )	20,633	2019-2024, Free full text, in the last five years, Humans, English	888
Google Scholar	Heart failure AND Sarcoidosis	64	2019-2024	28
Science Direct	Heart failure AND Sarcoidosis	16,058	2019-2024, English, Open access and Open archive	1,144

The records were filtered by title and abstract, which allowed for the elimination of articles that were irrelevant. The full-text articles were retrieved. In order to reduce the possibility of bias in this study, the articles that were successfully retrieved were examined using the proper quality assessment tools. The Scale for the Assessment of Non-Systematic Review Articles (SANRA) checklist was used to evaluate the narrative reviews. The systematic reviews and meta-analyses were assessed using the Assessment of Multiple Systematic Reviews (AMSTAR) checklist. Finally, we used the Newcastle-Ottawa Scale (NOS) to assess the cohort studies [9-11]. 

After the data was extracted, each co-author contributed equally to the completion of the retrieved data. The core outcomes, such as improvement in the cardiac function as measured by the ejection fraction and symptomatic improvement, were thoroughly examined in all the shortlisted publications.

Results

**Figure 1:**
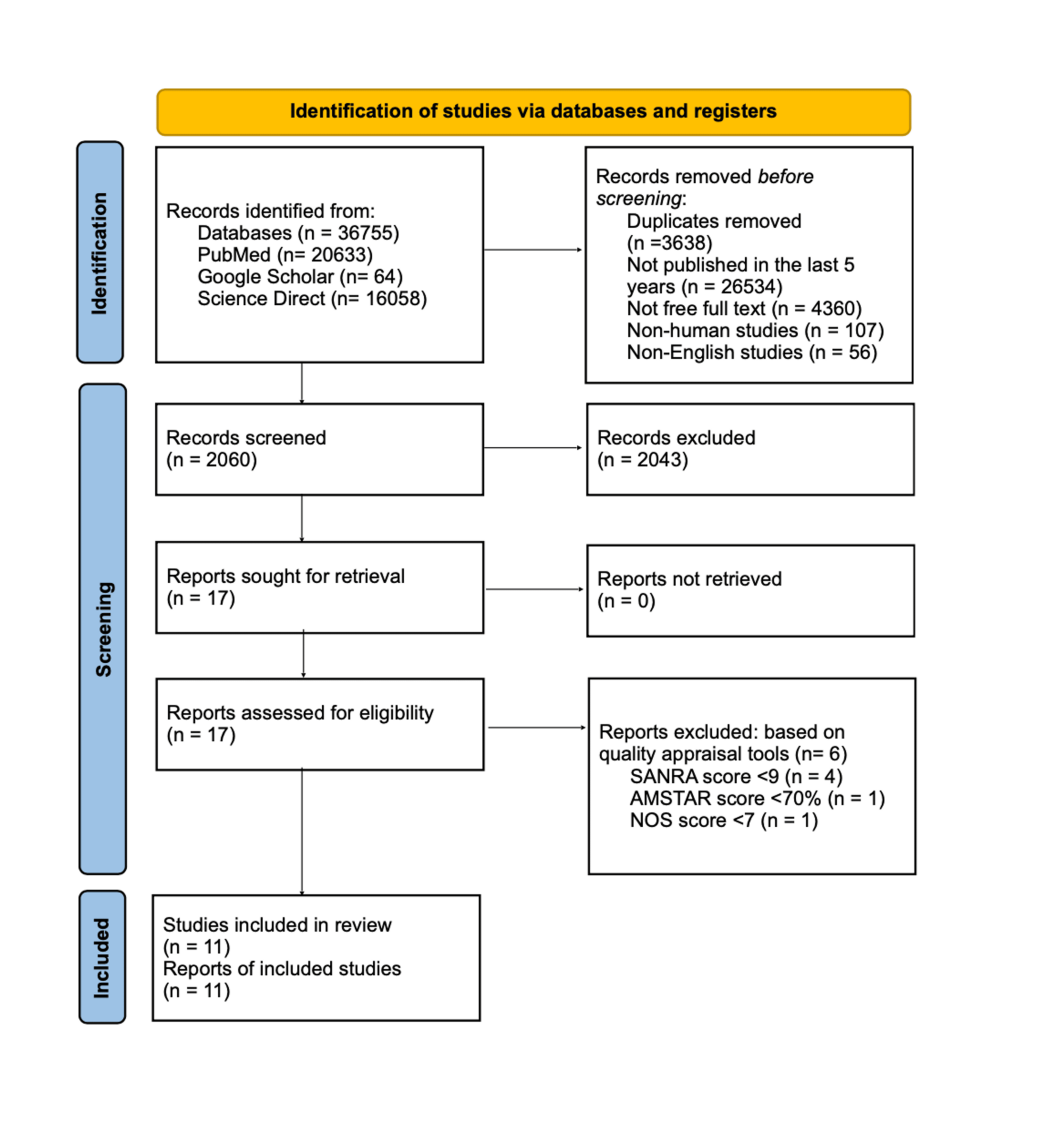
PRISMA chart PRISMA: Preferred Reporting Items for Systematic Reviews and Meta-analyses [8]. SANRA: Scale for the Assessment of Non-systematic Review; AMSTAR: Assessment of Multiple Systematic Reviews; NOS: Newcastle-Ottawa Scale

We obtained 36,755 articles from our initial search of PubMed, Google Scholar, and ScienceDirect. After applying the filters and removal of the duplicates using EndNote (version 21, Clarivate, Philadelphia, PA), the number of articles narrowed down to 2060. Following a screening process, out of 2060 articles, we discarded 2043 articles. After this process, 17 articles were left for retrieval. For a comprehensive quality/bias assessment utilizing standardized quality assessment methodologies, these 17 papers were included. Following a quality analysis, six papers were deemed unsuitable for inclusion in this systematic review, leaving the remaining 11 research studies to be reviewed. Figure 1 shows the PRISMA 2020 flow diagram [8].

Table 3 provides detailed insights into the quality assessment of the narrative reviews using the SANRA checklist.

**Table 3:** Evaluation results of narrative reviews using the SANRA 2 evaluation method Passing score: 9/12 SANRA 2: Scale for the Assessment of Non-systematic Review Articles 2 Reference: Baethge et al. [9]

First author, year	Justification of the article’s importance	Concrete aims or formulation of the question	Literature search described	Referencing	Scientific reasoning	Presentation of data	Sum	Pass/Fail
Eleftherios Markatis et al., 2020 [12]	2	1	0	2	2	2	9	Pass
Maria Giovanna Trivieri et al., 2020 [13]	2	1	0	2	2	2	9	Pass
Jukka Lehtonen et al., 2023 [6]	2	2	0	1	2	2	9	Pass
Richard K. Cheng et al., 2024 [14]	2	2	0	2	2	2	10	Pass
Pranav Mankad et al., 2019 [2]	1	1	0	2	2	2	8	Fail
Nisha A. Gilotra et al., 2022 [15]	2	2	0	2	2	2	10	Pass
Alessandro De Bortoli et al., 2023 [4]	1	2	1	1	1	2	8	Fail
Nisha Gilotra et al., 2020 [16]	1	2	0	2	1	2	8	Fail
Ilias C. Papanikolaou et al., 2022 [17]	1	1	0	2	2	1	7	Fail

Table 4 presents the evaluation results of the AMSTAR checklist for the systematic reviews.

**Table 4:** Evaluation results of systematic review articles using the AMSTAR checklist The passing score is >70% AMSTAR: Assessment of Multiple Systematic Reviews Reference: Shea et al. [10]

Checklist	Emanuele Bobbio et al., 2021 [18]	Julien Stievenart et al., 2022 [19]	Siavosh Fazelpour et al., 2021 [20]	Golnaz Roshankar et al., 2022 [21]
Was an "a priori" design provided?	Yes	Yes	Yes	Yes
Were there duplicates in study selection and data extraction?	Yes	Yes	Yes	Yes
Was a comprehensive literature search performed?	Yes	Yes	Yes	Yes
The status of publishing was considered as an inclusion criteria	Yes	Yes	No	Yes
Was a list of studies provided?	Yes	Yes	No	Yes
Were the characteristics of the included studies provided?	Yes	No	Yes	No
Was the scientific quality of the included study used appropriately in formulating conclusions?	Yes	Yes	Yes	Yes
Were the methods appropriate?	Yes	Yes	Yes	Yes
Was the likelihood of publication bias assessed?	No	No	No	No
Was the conflict of interest included?	Yes	Yes	No	Yes
Total Score	90%	80%	60%	80%
Pass/Fail	Pass	Pass	Fail	Pass

Lastly, we used the NOS to evaluate retrospective cohort research as described in Table 5.

**Table 5:** Details of the NOS assessment tool used in our study The passing score is 7/9. NOS: Newcastle-Ottawa Scale. Reference: Stang [11].

First author, Year	David G. Rosenthal et al., 2019 [22]	Matthew C. Baker et al., 2019 [23]	Rabea Asleh et al., 2022 [24]	Matthew T. McGoldrick et al., 2021 [25]
Representativeness of the exposed cohort	Yes	Yes	Yes	Yes
Selection of the non-exposed cohort	No	No	No	No
Ascertainment of exposure	Yes	Yes	Yes	Yes
Demonstrate that the outcome of interest was not existent at the start of the study	Yes	Yes	Yes	Yes
Comparability of the cohort-based design/analysis	Yes	Yes	No	Yes
Assessment of the outcome	Yes	Yes	Yes	Yes
Was the follow-up long enough?	Yes	Yes	Yes	Yes
Adequacy of follow-up cohorts	Yes	Yes	No	Yes
Pass/fail	Pass	Pass	Fail	Pass

Table 6 highlights the 11 studies that were used for this review based on satisfactory performance on their respective quality assessments.

**Table 6:** Details of the studies that passed the quality assessment and were selected for this systematic review

First author	Year	Type of study
Jukka Lehtonen et al. [6]	2023	Narrative review
Eleftherios Markatis et al. [12]	2020	Narrative review
Maria Giovanna Trivieri et al. [13]	2020	Narrative review
Richard K. Cheng et al. [14]	2024	Narrative review
Nisha A. Gilotra et al. [15]	2022	Narrative review
Emanuele Bobbio et al. [18]	2021	Systematic review
Julien Stievenart et al. [19]	2022	Systematic review
Golnaz Roshankar et al. [21]	2022	Systematic review
David G. Rosenthal et al. [22]	2019	Retrospective cohort research
Matthew C. Baker et al. [23]	2019	Retrospective cohort research
Matthew T. McGoldrick et al. [25]	2021	Retrospective cohort research

Our review highlights the essential roles of corticosteroids, immunosuppressants, and biologic drugs in treating CS. We examined 11 important studies that provide great insights into how well these treatments work. Corticosteroids were the go-to first-line therapy in every study, showing their crucial role in reducing inflammation and helping maintain heart function. Following corticosteroids, steroid-sparing medications like methotrexate were frequently used. Biologic therapies such as adalimumab and infliximab showed promise in targeting specific inflammatory pathways in difficult cases. Advanced imaging techniques, including cardiac MRI and FDG-PET, are essential for diagnosing, monitoring, and planning CS treatment. We explore deeper into these concepts to find an ideal therapeutic strategy for HF in CS. This review underscores the necessity for personalized care, continuous monitoring, and advanced treatment to improve patient outcomes.

Discussion

Role of Corticosteroids, Immunosuppressives, and Biological Agents

A comprehensive approach involving immunosuppressive agents that are aimed at reducing inflammation and preventing disease progression is involved in the treatment of CS. Corticosteroids like prednisone are usually initiated immediately after diagnosis to counteract myocardial scarring and preserve the left ventricular ejection fraction (LVEF). The optimal dose and duration of corticosteroids vary depending on the severity of the disease and the patient's response [12]. To reduce the long-term corticosteroid dependence and to avoid the adverse effects, steroid-sparing agents like methotrexate, azathioprine, and mycophenolate are typically employed as adjunctive therapies. These drugs provide stronger disease control when corticosteroids alone are not fully effective. A very crucial role is played by the biological agents, especially in refractory cases of CS where the corticosteroids and immunosuppressive agents have failed. Agents such as infliximab and adalimumab target specific inflammatory pathways involving tumor necrosis factor-alpha (TNF-α) to mitigate granuloma formation and disease progression [6,13,14]. Rituximab, which targets B cells' CD20, has shown promise in some CS patients and is now being investigated further for its potential uses [13].

Treatment Strategies and Their Impact on CS Remission

David G. Rosenthal et al. conducted a single-center retrospective cohort study with 28 individuals who were diagnosed with CS between January 2009 and November 2018. Following an initial high dosage of prednisone, patients with CS were treated with corticosteroid-sparing regimens primarily incorporating methotrexate and/or adalimumab. The primary goal of the study was to evaluate the effectiveness of sustaining illness remission by consecutive FDG-PET scans and clinical endpoints, such as VT and HF symptoms. The significance of continued immunosuppressive medication and close observation are highlighted by the findings, which demonstrated that stopping immunosuppression dramatically raised the likelihood of VT episodes, CS recurrence, and unfavorable clinical outcomes [22].

A retrospective study by Matthew C. Baker et al. examined 77 patients with CS from a cohort of 936 sarcoidosis patients seen between 2009 and 2018. The study was composed predominantly of Caucasians (66%), with a mean age of 55 years at diagnosis, and 39% were females. The study concluded that 32% of the participants had isolated CS and the typical initial presentations included heart block and tachyarrhythmias. FDG-PET and cardiac MRI were critical for establishing cardiac involvement. Prednisone (90%) and methotrexate (71%) were the common treatment modalities; TNF-α inhibitors like infliximab or adalimumab were used in 26% of the patients. This led to a significant improvement in image-based findings and a notable improvement in LVEF. The utility of TNF-α inhibitors in managing CS is highlighted by this study [23].

Comprehensive Management of Ventricular Arrhythmias and Advanced HF

CS management necessitates a customized strategy combining corticosteroids, steroid-sparing medications, and biological agents based on disease severity, treatment response, and patient-specific variables. In order to improve results for individuals with this difficult illness, ongoing research is still being done to investigate novel therapy alternatives and optimize existing treatment methods. Ventricular arrhythmias associated with CS are managed by antiarrhythmic drugs like amiodarone and sotalol, usually in association with corticosteroids during active inflammation [12]. These agents are critical to managing VT and atrial fibrillation and are used along with immunosuppressive drugs [6,14]. When it comes to treating severe cases of CS where HF has developed, angiotensin-converting enzyme (ACE) inhibitors and beta-blockers are essential [6,12]. Because of their dual effects in managing arrhythmia and heart failure, beta-blockers may occasionally be used. Optimizing cardiac performance and managing symptoms are the goals of using ACE inhibitors, angiotensin receptor blockers (ARBs), aldosterone antagonists, and sodium-glucose cotransporter-2 (SGLT-2) inhibitors [14]. ARBs, aldosterone antagonists, and diuretics are crucial in managing CS-related ventricular dysfunction. Beta-blockers, however, need careful use because of their ability to exacerbate atrioventricular block, necessitating pacemaker implantation [19,21]. Arrhythmias in CS can be controlled with a combination of antiarrhythmic drugs, catheter ablation for refractory cases, and implantable cardioverter-defibrillator (ICD) placement to prevent sudden cardiac death (SCD) [12-14]. In the context of heart transplantation (HTx), an extensive but personalized immunosuppressive regimen is important to balance graft survival and manage disease recurrence. Thymoglobulin or basiliximab are used as induction therapies to modulate the immune response, which leads to reduced rejection rates. Prednisolone and other immunosuppressants are utilized to optimize outcomes in long-term maintenance therapy [18,19].

Treatment strategies are influenced by multimodal imaging studies like cardiac PET and MRI scans. They help in evaluating the inflammation and scar burden [13]. Ventricular arrhythmias, refractory to antiarrhythmic drugs, may improve after the implementation of immunomodulators [6]. ICD helps in the prevention of sudden cardiac deaths, particularly in patients with advanced conduction defects [6,14]. The unpredictable nature of ventricular arrhythmias poses a serious threat in cases of CS; drugs like amiodarone and sotalol are the initial treatment options. In recurrent cases or in cases where there is intolerance to antiarrhythmic therapy, catheter ablation has been proven to be highly effective. Advanced atrioventricular blocks and bundle branch blocks may be triggered in cases of CS where the interventricular septum is involved. Placement of a permanent pacemaker may be required in such cases [15]. Cardiac MRI and FDG-PET scans are important in monitoring CS progression. TNF-α inhibitors are newer agents that have proven efficacious in reducing disease activity and improving patient outcomes [21].

A study by Matthew T. McGoldrick et al. used the organ procurement and transplantation network (OPTN) database to examine the outcomes of patients aged 18 years and older who underwent their HTx between January 1, 1999, and March 20, 2020 [25]. Patients were categorized into three groups based on their primary indication for transplant: restrictive cardiomyopathy due to cardiac sarcoidosis (RCM-sarcoidosis), restrictive cardiomyopathy due to other causes (RCM-other), and non-RCM indications. It was found that there was a significant increase in HTx for RCM-sarcoidosis over the study period, underscoring its rising prevalence. Patients in the RCM-sarcoidosis group were more likely to be younger, female, and chronically using steroids but less likely to have comorbidities such as diabetes or a history of tobacco use. The RCM-sarcoidosis group demonstrated superior long-term survival rates as compared to non-RCM patients after adjusting for relevant variables. Comparing RCM-sarcoidosis patients to their peers, secondary outcomes and causes of death analysis revealed unique disparities in morbidity and mortality patterns, including higher rates of fungal infections and other pulmonary causes. The study draws attention to the unique clinical features and shifting landscape of RCM-sarcoidosis in heart transplant recipients [25].

Treatment of patients suffering from advanced HF due to CS is challenging. There is no one-size-fits-all approach; the management has to be personalized. To tackle conduction abnormalities, cardiac resynchronization therapy (CRT) may be required in spite of mixed results, mainly due to widespread myocardial scarring. The use of ICDs to prevent SCD is common practice in the treatment [6,12,13]. In selected cases, a LVAD may be incorporated, after which HTx is the only definitive option for end-stage disease [6,14,15]. Evaluation of patients includes consideration of large-scale systemic involvement of the disease and immunosuppression such as diabetes or adrenal insufficiency [14]. Modern imaging modalities like cardiac MRI and FDG-PET are very important to diagnose and monitor CS. They allow us to examine the level of myocardial inflammation, scar burden, and response to treatment. These imaging techniques are therefore essential for pre-transplant assessment and post-transplant follow-up. Novel strategies in treatment are being studied to improve patient outcomes and to minimize the adverse effects of immunosuppression. Initiation of immunosuppressive therapy in the early stages based on imaging studies and the use of low-dose prednisone and methotrexate in combination with or without biologics like TNF-α antagonists are examples of the available measures undertaken to counteract CS. Table 7 provides a quick overview of the key findings of each study. The lack of standard protocols and validated biomarkers demonstrates the need for further clinical research and collaboration to refine these strategies and lay down clear guidelines for clinical practice [12-19].

**Table 7:** A summary of the key points of each study ACE inhibitors: angiotensin-converting enzyme inhibitors, ARBs: angiotensin receptor blockers, CS: cardiac sarcoidosis, FDG-PET: fluorodeoxyglucose positron emission tomography, HF: heart failure, HTx: heart transplantation, ICD: implantable cardioverter-defibrillator, LVEF: left ventricular ejection fraction, LVAD: left ventricular assist device, RCM: restrictive cardiomyopathy, SCD: sudden cardiac death, SGLT-2 inhibitors: sodium-glucose cotransporter-2 inhibitors, TNF-α: tumor necrosis factor-alpha, VT: ventricular tachycardia, MRI: magnetic resonance imaging

First author, Year	Conclusion(s)
Jukka Lehtonen et al., 2023 [6]	TNF-α inhibitors such as infliximab and adalimumab mitigate granuloma formation and progression of CS. Antiarrhythmic drugs like amiodarone and sotalol are used to manage ventricular arrhythmias and atrial fibrillations along with corticosteroids, which decrease inflammatory activity.
Eleftherios Markatis et al., 2020 [12]	Prednisone has proven to mitigate myocardial scarring and preserve LVEF. ACE inhibitors, ARBs, aldosterone antagonists, and SGLT-2 inhibitors are used to optimize cardiac performance and manage symptoms of HF. Ventricular arrhythmias are managed with the use of antiarrhythmic drugs like amiodarone and sotalol.
Maria Giovanna Trivieri et al., 2020 [13]	Rituximab is effective in controlling CS without any cardiac adverse effects. ICD helps prevent SCD in patients with advanced conduction defects. TNF-α inhibitors have a proven record of reducing disease activity and improving outcomes. Cardiac MRI and FDG-PET scans are essential for monitoring CS progression and treatment response.
Richard K. Cheng et al., 2024 [14]	Infliximab and adalimumab hamper the progression of CS. ACE inhibitors, ARBs, aldosterone antagonists, and SGLT-2 inhibitors are the cornerstones of the management of ventricular dysfunction. ICD helps in the prevention of sudden cardiac deaths. Cardiac MRI and FDG-PET scans are essential for monitoring CS progression.
Nisha A. Gilotra et al., 2022 [15]	In advanced atrioventricular blocks and bundle branch blocks, the use of a permanent pacemaker is key. Medically uncontrolled HF can be managed by the use of LVAD. End-stage disease may require a HTx.
Emanuele Bobbio et al., 2021 [18]	In HTx, the use of thymoglobulin or basiliximab helps in modulating the immune response and reduces the risk of rejection. Prednisolone and immunosuppressives can be utilized for their dual role in preventing graft rejection as well as control of systemic sarcoidosis.
Julien Stievenart et al., 2022 [19]	Beta-blockers, though they are helpful in HF, need to be used judiciously as they can exacerbate atrioventricular blocks necessitating a pacemaker implantation. Modern imaging modalities like cardiac MRI and FDG-PET are very important in helping clinicians learn about the response that the patient is showing to therapy.
Golnaz Roshankar et al., 2022 [21]	TNF-α inhibitors have proven efficacious in reducing disease activity and improving patient outcomes. Modern imaging modalities like cardiac MRI and FDG-PET are crucial for diagnosing and monitoring CS. Beta-blockers can be used to preserve LVEF, but they have the potential to cause life-threatening heart blocks.
David G. Rosenthal et al., 2019 [22]	Cessation of immunosuppressants leads to a rebound effect in the disease manifested by VT episodes and a drop in ventricular function; this highlights the importance of long-term relapse control using an effective regimen of immunosuppressives without compromising patient safety.
Matthew C. Baker et al., 2019 [23]	The effectiveness of TNF-α inhibitors, such as infliximab or adalimumab, in significantly improving image-based findings and LVEF is demonstrated.
Matthew T. McGoldrick et al., 2021 [25]	HTx in the RCM-sarcoidosis group has proven to show a superior long-term survival rate as compared to their non-RCM counterparts, hinting that HTx can be an effective option in end-stage disease.

Limitations

Treatment outcomes may be inconsistent due to the variability of corticosteroid dosing, durations, patient compliance, adverse effects, and the use of adjunctive therapies. Some of the content discussed has been derived from single-center studies; these results may not be generalizable to a broader population. The heterogeneous nature of CS can be demonstrated by its wide range of clinical manifestations and varied disease course, which might complicate the standard approach to therapy. Confounding factors such as other comorbidities may have played into the holistic patient management, which could have affected the study outcomes. Finally, the treatment protocols are subject to evolution over time, which may lead to some inconsistencies.

## Conclusions

CS is a serious presentation of sarcoidosis. It is characterized by the formation of non-necrotizing granulomas in the myocardial tissue. Effects of CS can primarily manifest in the form of arrhythmias and HF. Cardiac MRI, PET scan, and endomyocardial biopsy are used to diagnose CS and assess the level of inflammation and scar burden. HF in CS is managed by a personalized combination of conventional HF therapies like neurohormonal blockers and corticosteroids. Immunosuppressive agents and biological agents are used to reduce steroid dependence and to improve outcomes. Advanced and refractory HF can be managed by the use of LVAD. HTx is the ultimate solution to manage end-stage CS, with better outcomes of survival as compared to HTx due to other conventional cardiovascular causes. Appropriately tailored therapy can counteract myocardial scarring and improve the LVEF. Management of CS requires long-term therapy and monitoring, as very high disease recurrence is encountered with premature cessation of therapy. Many studies are in progress that aim to refine the treatment protocol and add more effective and safer treatment options. As CS is a very rare cause of HF, there is much need for international collaboration to set clear guidelines for treatment.
